# Enzyme‐Assisted Synthesis and In Vitro Characterization of Bifunctional PCSK9 Inhibitors

**DOI:** 10.1002/cbic.202500972

**Published:** 2026-04-08

**Authors:** Yuhui Zhang, Li Wang, Leo Corcilius, Amal K. Reji, Richard J. Payne, Bin Hong, Thomas Durek, Conan K. Wang, David J. Craik

**Affiliations:** ^1^ Institute for Molecular Bioscience The University of Queensland Brisbane Australia; ^2^ Institute of Medicinal Biotechnology Chinese Academy of Medical Science & Peking Union Medical College Beijing China; ^3^ School of Chemistry The University of Sydney Sydney Australia

**Keywords:** drug design, hypercholesterolemia, LYTAC, peptide

## Abstract

Proprotein convertase subtilisin/kexin type 9 (PCSK9) is a well‐established target for lowering cholesterol and is abundantly present in the extracellular space. Inhibitors of PCSK9 have achieved marked success in the clinic, but an alternative strategy for therapeutic modulation is emerging through the degradation of PCSK9. This novel strategy has been enabled by the identification of cell surface receptors such as the asialoglycoprotein receptor (ASGPR), which mediates the lysosomal degradation of extracellular ligands. Given the importance of this therapeutic mechanism, we investigated the synthesis of bifunctional molecules comprising Tri‐GalNAc (an ASGPR binder) with a peptide inhibitor we previously reported. In addition to chemical synthesis, we report a novel method for the production of Tri‐GalNAc‐conjugated peptides, involving the use of enzymatically mediated ligation postsynthesis. We demonstrate that both the synthetic constructs and chemoenzymatic constructs have the intended structures and in vitro activities. While these molecules did not show cellular activities, the chemical and biochemical methods reported here could be broadly applied to the construction of LYTACs in general. One significant challenge that this work overcomes is the C‐terminal attachment of Tri‐GalNAc, which remains hitherto a difficult experimental task for not only peptides but also larger biologics in particular.

## Graphical abstract

Proprotein convertase subtilisin/kexin type 9 (PCSK9) is an important therapeutic target for lowering cholesterol. Here, we report the assembly of a bifunctional molecule combining a PCSK9‐binding peptide with an asialoglycoprotein receptor (ASGPR) ligand. In addition to solid‐phase peptide synthesis, a chemoenzymatic ligation strategy enabled efficient C‐terminal Tri‐GalNAc attachment. The constructs were structurally validated and retained in vitro activity toward PCSK9 and ASGPR.

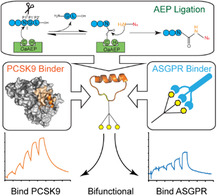

## 1 Introduction

Hypercholesterolemia is one of the most significant risk factors for cardiovascular disease, a leading cause of morbidity and mortality worldwide [1]. Over the past few decades, statins have been the most widely used drugs for lowering cholesterol, becoming some of the best‐selling medications in history, and highlighting the high demand for cholesterol‐lowering therapies [2]. Nevertheless, a substantial proportion of patients fail to achieve adequate cholesterol reduction with statins, indicating the need for new therapeutic approaches [3].

Meanwhile, proprotein convertase subtilisin/kexin type 9 (PCSK9) was identified as a novel regulator of cholesterol through studies on familial hypercholesterolemia [4]. PCSK9 reduces the levels of low‐density lipoprotein (LDL) receptors (LDLR) on the hepatocyte membrane, thereby impairing the clearance of LDL cholesterol [5]. Inhibition of PCSK9 enhances LDLR abundance, leading to improved cholesterol catabolism. Consequently, PCSK9 has been established as a clinically validated therapeutic target for hypercholesterolemia, as demonstrated by the approval of two monoclonal antibodies and a small interfering RNA (siRNA) therapy, all of which have shown high cholesterol‐lowering efficacy and favorable safety profiles [6, 7]. Even so, there remains scope for the development of new drug candidates targeting PCSK9, since the current therapeutics are expensive [8] and require administration by injection, which limits patient accessibility and adherence [9].

Peptides are promising modalities for targeting PCSK9 since they have the potential for better cost‐effectiveness and oral bioavailability [10, 11]. Owing to their intermediate size, they are suitable for modulating protein–protein interactions, such as the interaction between PCSK9 and LDLR, while remaining amenable to efficient chemical synthesis and structural optimization. A representative example is Pep2−8, a linear PCSK9 inhibitory peptide identified through phage display screening [12]. Pep2−8 is advantageous as it is a small, readily synthesized peptide that exhibits submicromolar inhibitory activity (*IC*_50_ = 0.44 µM), providing substantial potential for further optimization. Various strategies have been explored to enhance its properties, including computational design [13], stapling [14], cyclization [15], and dendrimer conjugation [16] to improve binding affinity, as well as lipidation [17], and nanoparticle formation [18, 19] to increase in vivo stability. Among the Pep2−8 analogs, P9‐mut exhibited one of the highest potencies, with a binding affinity of *K*_D_ = 2 nM to PCSK9. Its design involved two steps: initial bioactive cyclization to enhance activity [15], followed by alanine scan and site‐specific mutations to improve both affinity and serum stability [17]. P9‐mut was further optimized through lipidation [17], which enhanced its cholesterol‐lowering efficacy in mice to a level approaching that of an antibody, suggesting its potential as a therapeutic scaffold.

An alternative strategy to enhance the in vivo efficacy of P9‐mut is to convert it into a degrader. Unlike inhibitory peptides, which eventually dissociate from PCSK9 and lose their antagonistic activity, degraders irreversibly eliminate the target protein once bound. In support of this hypothesis, the siRNA therapeutic, which acts by downregulating PCSK9 expression, has a substantially longer‐acting time than anti‐PCSK9 antibodies [20].

**FIGURE 1:**
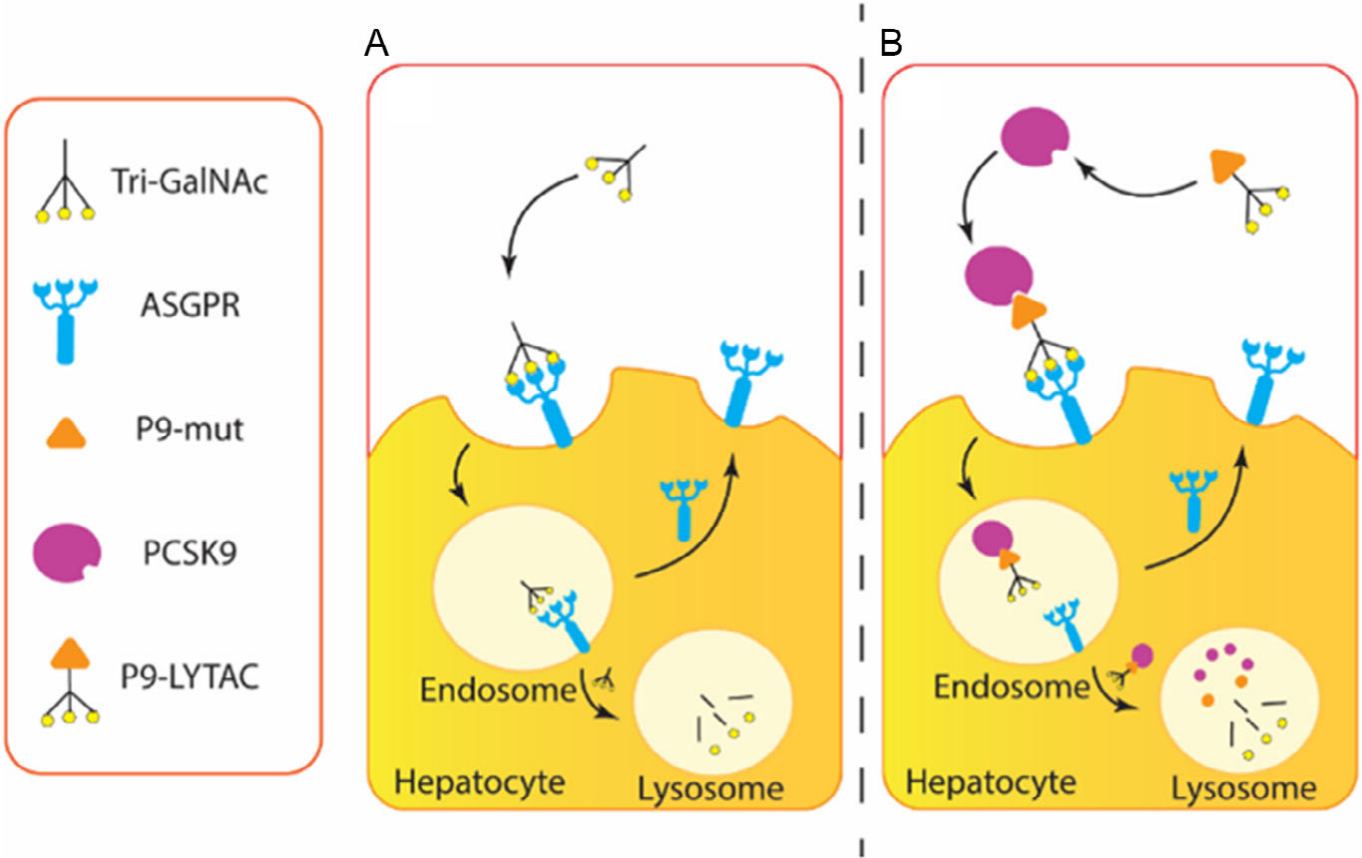
Mechanism of ASGPR‐induced degradation and P9‐LYTAC. (A) Tri‐GalNAc binds to ASGPR on the hepatocyte surface and then internalizes to the lysosome for degradation; ASGPR will be recycled to the cell surface. (B) P9‐LYTAC binds to PCSK9 and ASGPR at the same time, and the complex of PCSK9 and P9‐LYTACs will be internalized to the lysosome for degradation.

Targeted protein degradation (TPD) is an emerging drug development strategy [21]. However, most TPD strategies rely on the ubiquitin–proteasome pathway, which is not suitable for PCSK9, as they target intracellular or membrane proteins, whereas PCSK9 is a secreted extracellular protein. In 2020, the LYTAC was first reported by Bertozzi's research group [22]. It utilizes lysosome‐targeting receptors (LTRs) on the cell surface to shuttle the target protein to lysosome for degradation. Therefore, the LYTAC strategy is suitable for developing degraders against PCSK9. The first LTR exploited for LYTACs was cation‐independent mannose‐6‐phosphate receptor (CI‐M6PR), which recognizes mannose‐6‐phosphonate (M6Pn) as its ligand [22]. The second identified LTR was asialoglycoprotein receptor (ASGPR), which recognizes N‐acetylgalactosamine (GalNAc) as its ligand [23, 24, 25]. The second‐generation ASGPR–LYTACs have better tissue specificity than the first‐generation CI‐M6Pn–LYTACs because ASGPR is specifically expressed in the liver, while CI‐M6PR is ubiquitously expressed in many tissues [22]. This enhanced specificity may reduce systemic toxicity and broaden the therapeutic window [23, 26, 27]. The liver is also where PCSK9 is produced, which thus could enhance the efficiency of PCSK9 degradation. LYTACs consist of a target‐binding domain and a LTR domain. For ASGPR–LYTACs, one well‐studied lysosome‐targeting moiety is the carbohydrate Tri‐GalNAc, which has been shown to have a high affinity for ASGPR [23, 25, 28, 29] (Figure 1A). In addition, GalNAc–ASGPR is a well‐defined delivery system that has been successfully used for delivering siRNA in a clinical setting [30].

To date, only a few studies have identified PCSK9 degraders. Apart from the two reports on small‐molecule PCSK9 degraders [31, 32], only one study has described the development of a peptide‐based PCSK9 degrader [28]. In this study, the authors employed the same strategy as LYTACs, developing heterobifunctional peptides that bind both PCSK9 and ASGPR, which demonstrated PCSK9 degradation activity in mouse models. This finding demonstrated the feasibility of this approach, and further studies are warranted to strengthen it. However, the molecule reported in that study was synthesized via chemical ligation of the component parts, which may pose limitations for large‐scale manufacturing and cost‐effective production. Therefore, it is important to explore alternative designs that can be prepared using more economical and scalable chemoenzymatic synthetic strategies for PCSK9 degradation.

In this study, Tri‐GalNAc was conjugated to P9‐mut to convert the peptide into bifunctional molecules. The resulting analogs are referred to as P9‐LYTACs (Figure 1B). P9‐LYTACs were synthesized using either chemical or chemoenzymatic approaches, and they exhibited submicromolar binding affinity toward ASGPR while retaining PCSK9 binding with only an approximately 10‐fold reduction in affinity.

## 2 Results and Discussion

### 2.1 Design of P9‐LYTACs

**FIGURE 2:**
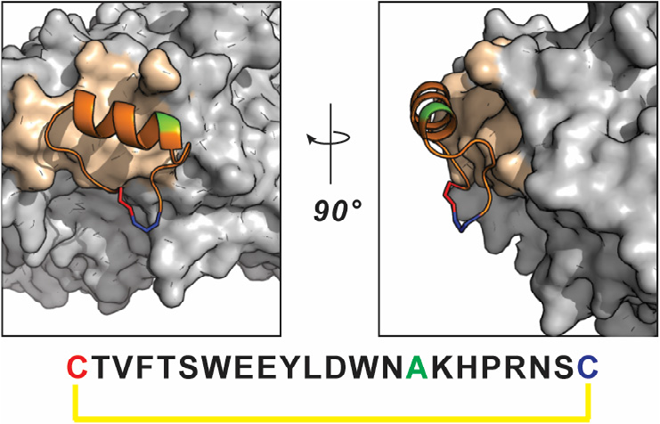
The modeled structure of P9‐mut bound to PCSK9. P9‐mut (orange) binds to PCSK9 (gray), and the binding interface is colored beige (PDB 2PMW). The N‐terminal Cys of P9‐mut is colored in red, the C‐terminal Cys is colored in blue, and Ala15 is colored in green.

To design a heterobifunctional PCSK9 ligand, it is crucial to preserve the original activity of each monovalent building block. Tri‐GalNAc has been shown to retain binding affinity to ASGPR by incorporating a short caprylic acid linker between its cargo and itself [23, 28]. Therefore, we focused on exploring the effect of linker attachment on P9‐mut, optimizing for binding activity against PCSK9. We chose three potential linker attachment sites on the peptide: a) the N‐terminus, b) the C‐terminus, or c) the side chain of Ala15. According to our previous study [17], the addition of extra functionalities to either the N‐ or the C‐terminus via polyethylene glycol (PEG) linkers had only minor effects on PCSK9 affinity, suggesting that these changes are well tolerated. Ala15 was selected for two reasons: (1) In an alanine scan of P9−38, the parent peptide to P9‐mut, mutation of N15 did not affect the affinity of the peptide, suggesting that Position 15 of both P9−38 and P9‐mut is not involved in the direct binding to PCSK9 [15]; and (2) an in silico modeled structure of P9‐mut bound to PCSK9 showed that this position faces away from the binding interface (Figure 2).

### 2.2 Chemical Synthesis of P9‐LYTAC Precursors

**FIGURE 3:**
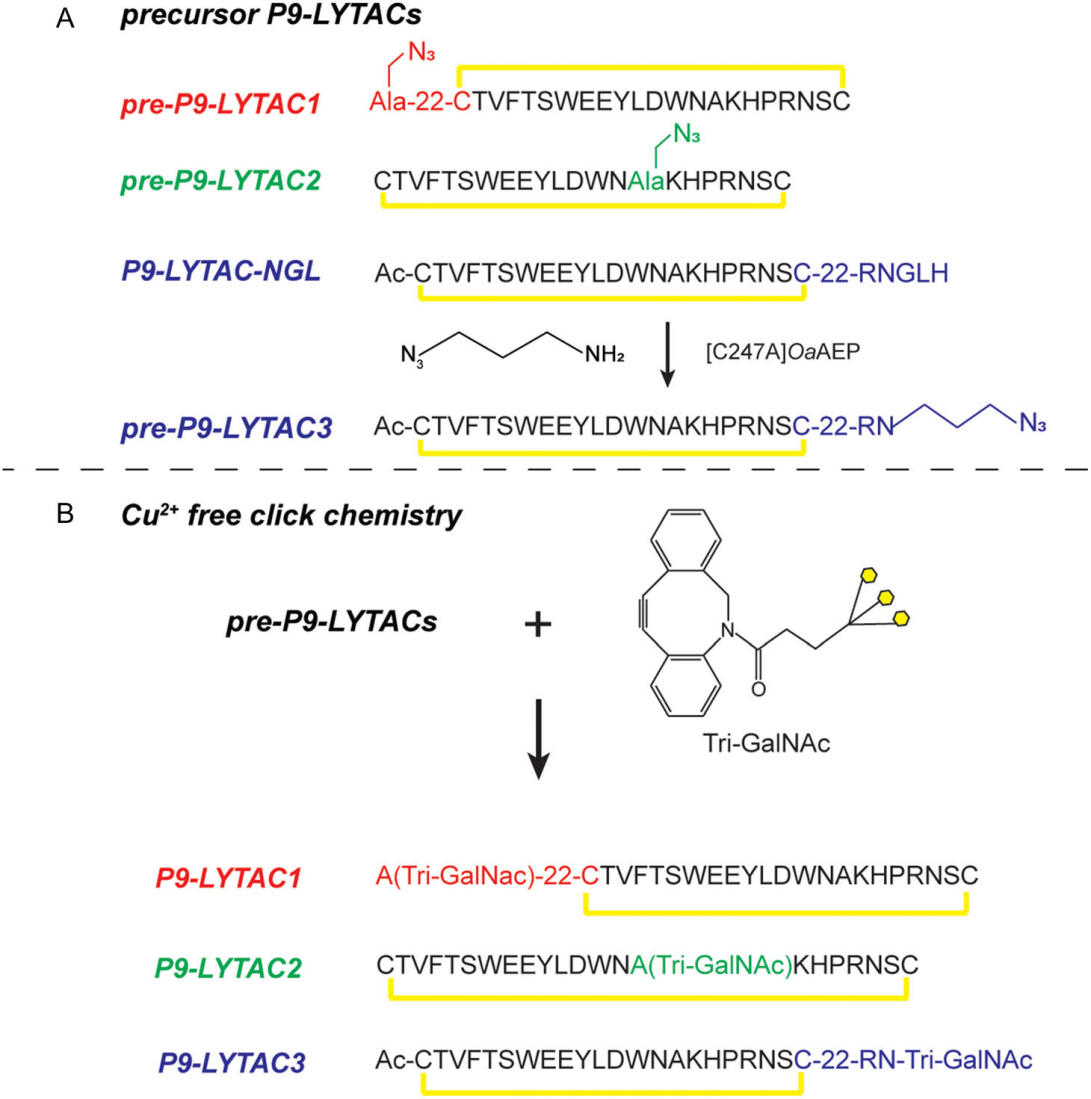
The design and synthesis strategy of P9‐LYTACs. (A) The design of the precursor of P9‐LYTACs, 22 denotes a two‐unit PEG2 linker. (B) DBCO–Tri‐GalNAc selectively ligates to the azide on the precursors of P9‐LYTACs via strain‐promoted azide–alkyne cycloaddition.

P9‐LYTACs consist of P9‐mut and Tri‐GalNAc. We decided to conjugate these two moieties via Cu^2+^ free click chemistry (Figure 3B) since it is a well‐established method for conjugating Tri‐GalNAc (Figure S1A) to biomolecules [23]. The precursor P9‐LYTAC peptides with an azido group at the desired ligation site were synthesized by Fmoc‐SPPS. Specifically, for the P9‐LYTAC1 precursor peptide, an azido‐alanine was incorporated at the N‐terminus of P9‐mut along with two PEG2 linkers during SPPS (Figure 3A). Similarly, azido‐alanine replaced the Ala15 of the P9‐mut to make the precursor peptide of P9‐LYTAC2 (Figure 3A).

### 2.3 Enzyme‐Assisted Synthesis of P9‐LYTAC3 Precursor

**FIGURE 4:**
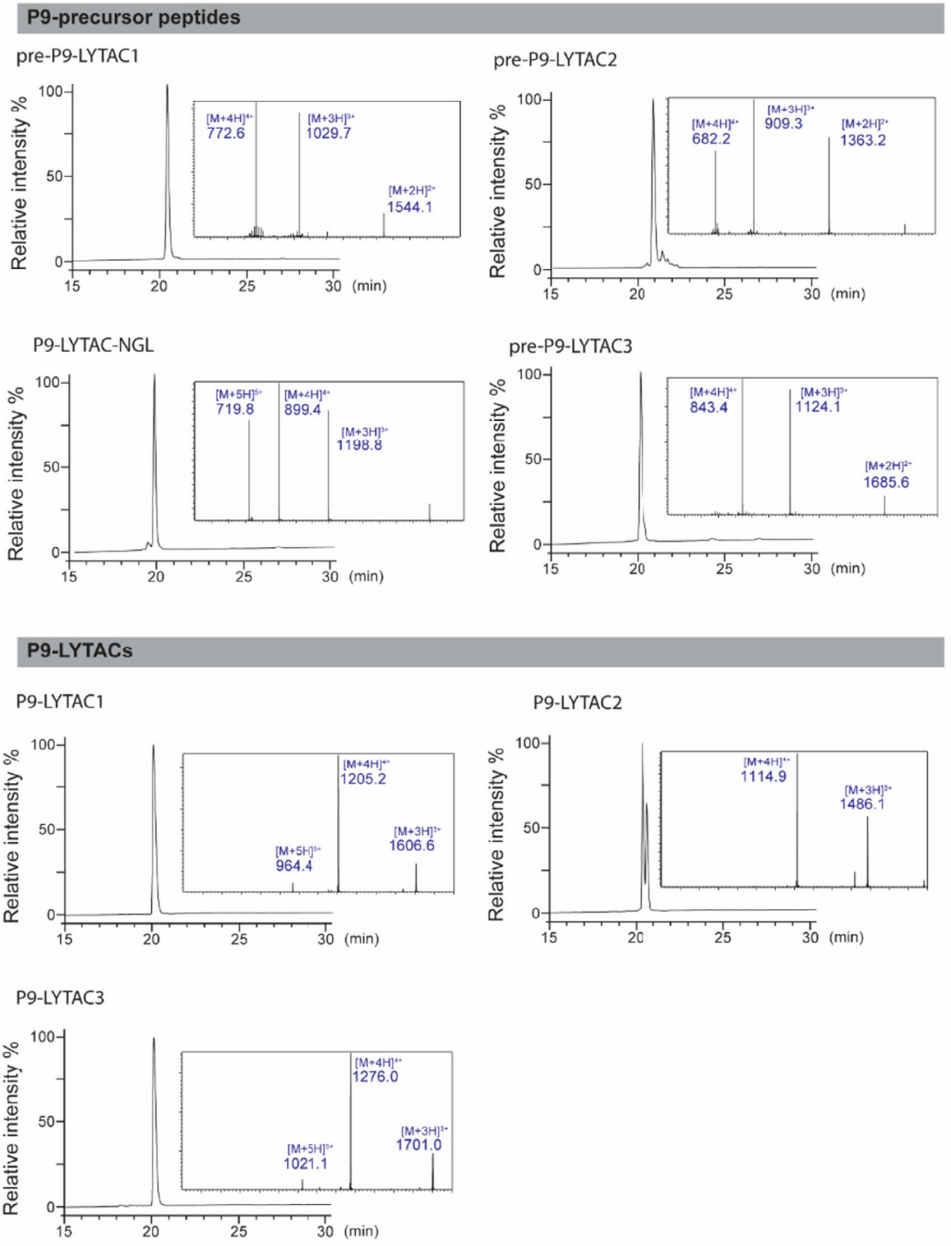
Analytical RP‐HPLC traces and ESI‐MS characterization of peptides used in this study. Precursor peptides were purified to >90% purity, and P9‐LYTACs were purified to >95% purity based on 214 nm absorbance. A linear 2%/min gradient was used from 0.05% v/v TFA in water to 0.05% v/v TFA in 80% v/v acetonitrile. The ESI‐MS spectra show [M + 4H]^4+^, [M + 3H]^3+^, and/or [M + 2H]^2+^ ions of each peptide; their theoretical and calculated masses are shown in Table S1.

To synthesize the P9‐LYTAC3 precursor, a P9‐mut analog (P9‐LYTAC‐NGL) was first prepared. This construct contained a C‐terminal RNGLH sequence (an asparaginylendoproteinase (AEP), recognition motif), followed by two PEG2 units as a linker, and the P9‐mut sequence with an acetylated N‐terminus. After cleavage and purification, AEP was used to install an azido amine at the C‐terminus of the Asn within the recognition site (Figure 3A) [33]. The ligation was performed with an excess (1000 equivalents) of 3‐azido‐1‐propanamine and 0.005 equivalents of [C247A]*Oa*AEP in 100 mM HEPES, pH 7.5. The reaction was completed within 12 h, as no starting material (P9‐LYTAC‐NGL) was detected by mass spectrometry analysis. After reverse phase high‐performance liquid chromatography (RP‐HPLC) purification to >95% purity (Figure 4), the ligated pre‐P9‐LYTAC3 was then lyophilized, and the isolated yield was approximately 70% (w/w, relative to P9‐LYTAC‐NGL). By using this method, no unnatural amino acids such as azido‐alanine were required during peptide synthesis (the PEG linkers could be replaced with peptide linkers, and the N‐terminal amine could be neutralized via site‐selective transamination [34, 35]). Therefore, LYTAC3 could potentially be manufactured via recombinant expression in a more environmentally friendly and cost‐effective manner.

### 2.4 Click Ligation of P9‐LYTAC Precursors to Tri‐GalNAc

For the click reaction, the P9‐LYTAC precursors were reacted with DBCO–Tri‐GalNAc at a 1:1 molar ratio in phosphate buffered saline (PBS) (Figure 3B). The reactions were complete within 2 h, as no precursor peptides were detected by mass spectrometry analysis. P9‐LYTACs were then purified by RP‐HPLC to a final purity of at least 95% (Figure 4). Based on the weight of the dried product, the isolated yield of P9‐LYTACs from the click reaction was approximately 50% (w/w, relative to the dried precursors). Notably, two peaks were observed in the HPLC of P9‐LYTAC2 due to the formation of regioisomers (1,4‐ or 1,5‐disubstituted triazoles) from the Cu^2+^ free click chemistry [36]. The double peak was not observed for LYTAC1 and P9‐LYTAC3 (Figure 4). This may be due to the fact that the ligation site of P9‐LYTAC2 is within its constrained backbone and lacks a linker, which might explain why the isomerization had a greater impact on its physical properties compared to P9‐LYTAC1 and P9‐LYTAC3, whose ligation sites are located at the flexible termini (Figure 3B).

### 2.5 Structural Analysis of P9‐LYTACs

**FIGURE 5:**
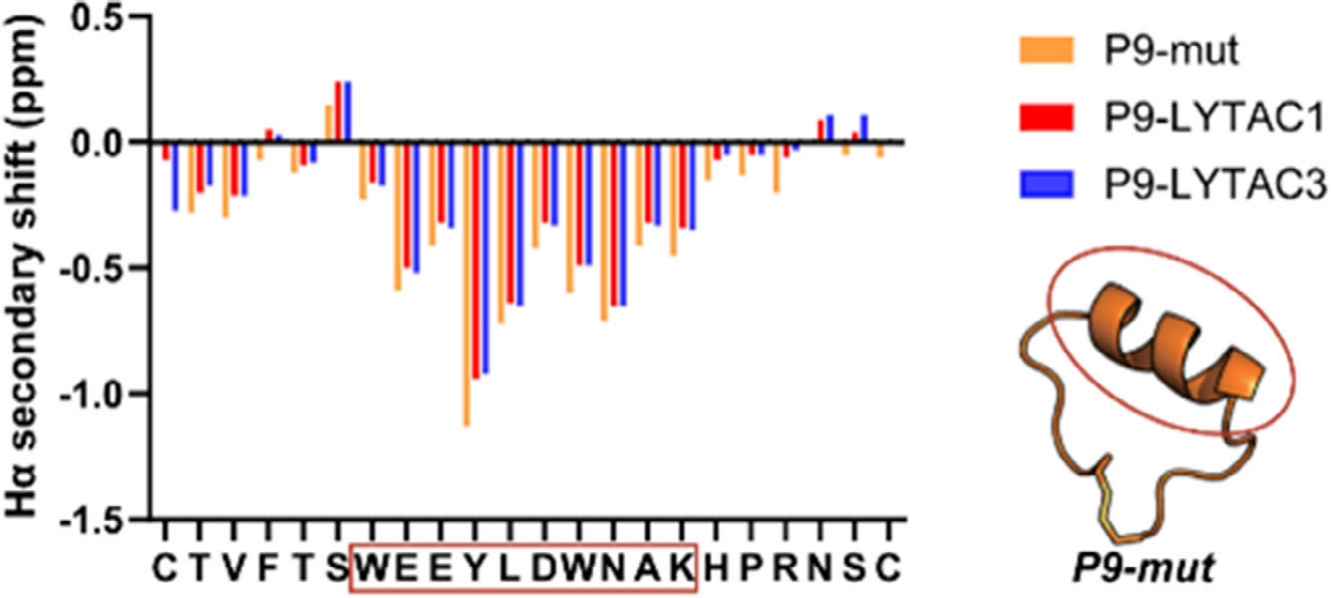
Hα secondary NMR chemical shifts of P9‐LYTACs and P9‐mut measured in 30% v/v CD_3_CN. The parent peptide P9‐mut is presented in orange, and P9‐LYTAC1 and P9‐LYTAC3 are presented in red and blue, respectively. The red bracket presents the helical region of P9‐mut which is also preserved in the LYTAC analogs.

The effect of Tri‐GalNAc fusion on the structure of the P9‐mut moiety was evaluated by NMR. ^1^H, total correlation spectroscopy (TOCSY), and nuclear Overhauser effect spectroscopy (NOESY) NMR spectra were recorded for P9‐LYTACs in H_2_O:CD_3_CN (7:3, v/v) at 298 K. The secondary Hα chemical shifts of P9‐LYTAC1 and P9‐LYTAC3 were similar to those of P9‐mut, indicating that fusion of Tri‐GalNAc did not affect the overall structure of the parent peptide (Figure 5) [37]. The chemical shift assignment of P9‐LYTAC2 was challenging due to the presence of regioisomers, as evidenced by peak splitting observed in the one‐dimensional (1D) spectra (Figure S2).

### 2.6 Biophysical Examination of the Bifunctional Binding Activities of P9‐LYTACs

**FIGURE 6:**
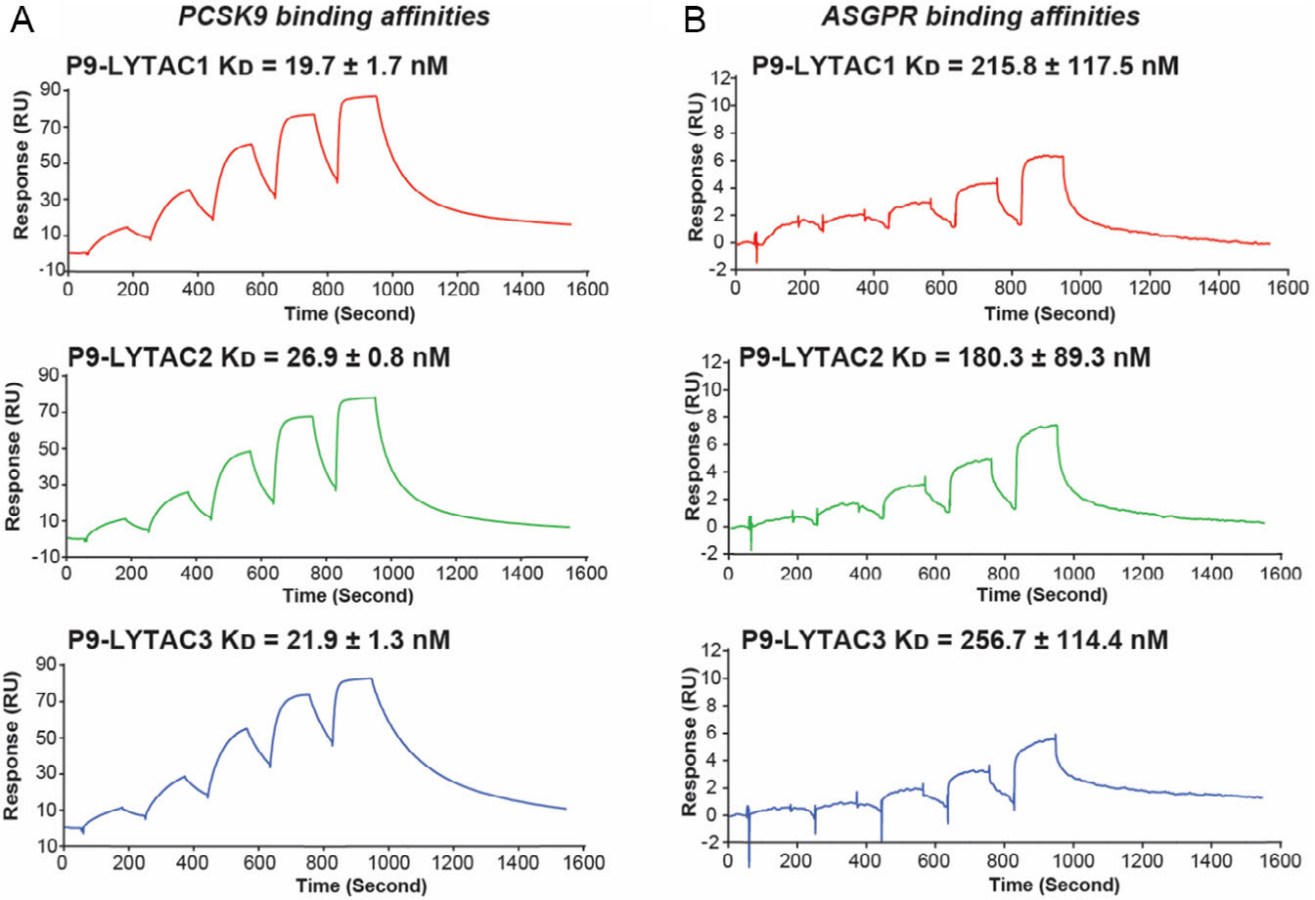
Biophysical measurement of binding affinities of P9‐LYTACs to PCSK9 and ASGPR. (A) The binding affinities of P9‐LYTACs to PCSK9 were measured by SPR by using a single‐cycle kinetic. The derived dissociation constants (*K*_D_) as average ± SD are shown above the representative sensorgrams of each P9‐LYTACs, respectively. (B) The binding affinities of P9‐LYTACs to ASGPR were measured by SPR by using a single‐cycle kinetic. The derived dissociation constants (*K*_D_) as average ± SD are shown above the representative sensorgrams of each P9‐LYTACs, respectively. All experiments were performed in triplicate.

Surface plasmon resonance (SPR) was used to investigate the binding affinities of P9‐LYTACs to PCSK9 and ASGPR. To measure the affinity for PCSK9, biotinylated PCSK9 was immobilized on a streptavidin sensor (SA) sensor chip, and single‐cycle kinetic experiments were performed (analyte concentration: 4, 16, 64, 256, 1024 nM). The PCSK9‐binding affinities of the peptides modified at N‐ or C‐terminus (P9‐LYTAC1: 19.7 ± 1.7 nM and P9‐LYTAC3: 21.9 ± 1.3 nM) were similar to each other, which was consistent with our previous study reporting on the binding affinities to PCSK9 of terminally modified P9‐mut with PEG linkers and lipid groups [17]. The binding affinity of P9‐LYTAC2 was 26.9 ± 0.8 nM, which was only slightly weaker than that of the termini‐modified peptides, showing that modification of Ala15 is well tolerated. Overall, all P9‐LYTACs exhibited binding affinities of ∼20 nM toward PCSK9 (Figure 6A). Although their affinities decrease upon conjugation to Tri‐GalNAc or termini extension compared to the native peptide P9‐mut (2.1 ± 0.5 nM), they are similar to the PCSK9 affinity of P9‐albN2 (29.9 ± 7.1 nM), an analog of P9‐mut, which we showed was active in the mouse model of hyperlipidemia [17].

SPR was also performed to measure the binding affinities of P9‐LYTACs to the ASGPR immobilized on a SA chip. The single‐cycle kinetic method was the same as in the PCSK9 SPR experiment. All P9‐LYTACs have approximately 200 nM binding affinities to ASGPR (Figure 6B). However, the maximal response units (*R*_max_) observed in this experiment were low (∼6 RU, compared to the theoretical value of ∼100 RU, as calculated below). This led to greater variability in the fitted affinity curves (Figure S3), albeit with slight differences in RU, and resulted in a high standard deviation of the measured *K*_D_ values (Figure 6B). The absence of detectable binding for P9‐mut confirmed that ASGPR recognition was mediated by the Tri‐GalNAc moiety in P9‐LYTACs (Figure S4A). We also tested the binding affinity of Propylamine–Tri‐GalNAc (Figure S1B) using the same conditions. However, it did not show apparent binding until the concentration reached 1 µM (Figure S4B). It could be because this SPR setup was not suitable for evaluating the binding of Tri‐GalNAc to ASGPR. Overall, the evaluated binding affinities of P9‐LYTACs to ASGPR were approximately 100‐fold lower than that of Tri‐GalNAc, as reported in other studies (reported *K*_D_ or *K*_i_ is approximately 3 nM) [28, 38]. Nevertheless, the results indicate that P9‐LYTACs bind to ASGPR with submicromolar affinities.

### 2.7 Serum Stability of P9‐LYTACs

**FIGURE 7:**
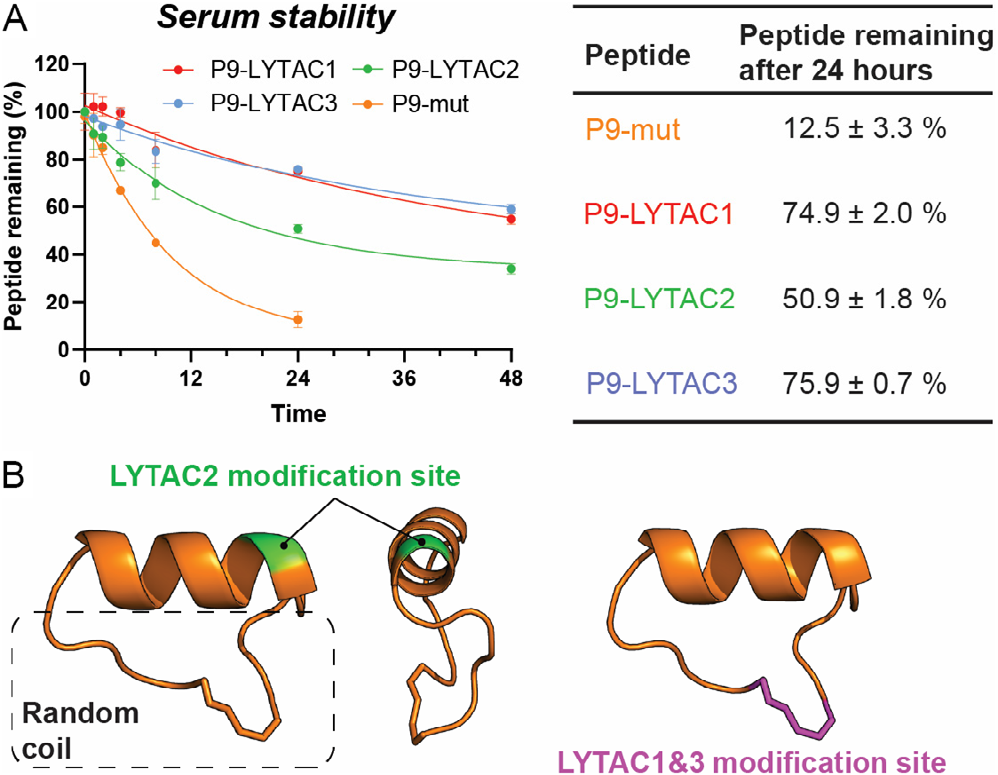
Serum stability evaluation of P9‐LYTACs. (A) Serum stabilities of P9‐LYTACs and P9‐mut. The stability assay was performed for 48 h for P9‐LYTACs and 24 h for P9‐mut. Data points are the average ± SD of triplicate measurements. The values of peptides remaining after 24 h of serum incubation are compared in the table on the right (*n* = 3). (B) The modification site of LYTAC2 is colored in green and the modification sites of LYTAC1 and LYTAC3 are colored in purple.

It was crucial to evaluate the serum half‐lives of the peptides since a short serum half‐life could significantly diminish their activity in vivo. Based on the HPLC analysis of the sample treated with serum, all P9‐LYTACs showed higher half‐lives than the parent peptide, P9‐mut (Figure 7A). These results might be due to the bulky Tri‐GalNAc group introducing steric hindrance between serum proteases and the peptides. The extent of steric protection may also depend on the local structural rigidity of the peptide, which can influence its inherent resistance to proteolytic attack (Figure 7B). The modification site of P9‐LYTAC2 was placed within the structured helical segment. On the other hand, the modification sites of P9‐LYTAC1 and P9‐LYTAC3 were at the termini within a flexible random coil region of the peptide. Overall, the enhanced serum stability of P9‐LYTACs supports their suitability for evaluation in cell‐based assays, which typically involve an incubation period of ∼24 h.

This interesting improvement in serum stability indicates that adding a bulky moiety to the peptide could potentially introduce steric hindrance between the peptide and proteases. Moreover, P9‐LYTAC1 and P9‐LYTAC3 with modifications near the flexible linker have better serum stability than P9‐LYTAC2, which has a modification within the rigid helical structure that is far from the flexible part of the peptide. These results suggest that positioning the bulky moiety closer to protease‐susceptible regions enhances steric shielding, thereby improving serum stability. A similar scenario is also observed in nature; for example, glycosylation can protect the iron transporter ZIP14 from proteolytic degradation [39].

### 2.8 Cellular PCSK9 Degradation Activity Evaluation of P9‐LYTACs

To evaluate the PCSK9 degradation activity of P9‐LYTACs, the levels of the secreted PCSK9 were quantified by enzyme‐linked immunosorbent assay (ELISA) (Figure S5A). The levels of PCSK9, secreted PCSK9 (sPCSK9), and LDLR of HepG2 cells, were measured by Western blot (Figure S5B). The HepG2 cells were treated with 20 and 200 nM of P9‐LYTACs for 24 h, as well as with P9‐mut (20 and 200 nM) as a negative control and the PCSK9 expression inhibitor C5 (10 µM) as a positive control (Figure S6) [40]. Unfortunately, the P9‐LYTACs did not show PCSK9 degradation activity in this cell assay; the secreted PCSK9 levels of the cells treated with P9‐LYTACs were equivalent to those in cells treated with PBS or P9‐mut (Figure S5A). As shown in Figure S5B, P9‐LYTACs still had LDLR recovery activity, indicating that the lack of PCSK9 degradation was not due to reduced binding affinity for PCSK9.

The result was unexpected, since Tri‐GalNAc has been confirmed as a potent binder to ASGPR that can efficiently transport biomolecules and nanoparticles to lysosomes [41], and HepG2 is a liver‐derived cell line reported to express ASGPR [42]. The deficiency of P9‐LYTACs may be attributed to their inability to simultaneously engage PCSK9 and ASGPR, or to insufficient functional expression of ASGPR in the cells used for the assay.

## 3 Conclusion

In this study, we combined the PCSK9 inhibitory peptide P9‐mut with Tri‐GalNAc to make bivalent P9‐LYTACs, which could potentially degrade PCSK9. The P9‐LYTACs were prepared through both chemical and enzymatic site‐specific modifications of P9‐mut. The bivalent activities of P9‐LYTACs were confirmed by SPR; all three analogs showed similar high‐affinity binding to PCSK9 (∼20 nM) and ASGPR (∼200 nM). The P9‐LYTACs also exhibited greater serum stability (with over 50% remaining after 48 h of incubation) compared to their parent peptide, P9‐mut, indicating that the addition of bulky moieties can enhance the serum stability of the peptide. Together, the in vitro experiments demonstrate that the chemical and biochemical methods reported here could successfully make bifunctional peptides comprising a PCSK9‐targeting component and an ASGPR binder.

Notably, the successful application of AEP‐mediated enzymatic ligation provides a practical strategy for constructing similar chimeric biomolecule degraders. In this approach, the targeting biomolecule can be produced recombinantly and subsequently site‐specifically labelled through a combination of AEP ligation and click chemistry. Compared with existing site‐specific conjugation platforms for LYTAC generation, such as SMARTag [43], AEP requires a shorter recognition sequence and leaves a smaller ligation scar, thereby minimizing structural perturbation to the parent molecule [44]. In addition, a previous study reported a site‐specifically conjugated PCSK9 antibody for targeted PCSK9 degradation [45], in which an endoglycosidase‐based conjugation strategy was employed [46]. However, this approach is inherently limited to glycosylated proteins. In contrast, AEP recognizes a short peptide motif that can be readily incorporated into a wide range of recombinantly expressed proteins or peptides, enabling broader applicability and greater flexibility in degrader design.

A key hypothesis of this study was that the PCSK9 degrader would exhibit greater cholesterol‐lowering activity than the inhibitor. During this research, a similar study was published that developed heterobifunctional PCSK9 degraders by conjugating a binder to ASGPR ligands [28]. Although the degrader demonstrated effective PCSK9 degradation, the study noted that there was no improvement in cholesterol‐lowering activity compared to the inhibitor alone. The result suggested that this outcome may be attributed to rapid ASGPR‐mediated clearance of the degrader, which could eliminate the molecule before sufficient target engagement, as well as the “hook effect” associated with the bifunctional design [47]. One potential strategy to address this limitation is the application of the recently developed nano‐LYTAC platform, which does not rely on LTRs. Similar to conventional LYTAC systems, nano‐LYTACs are capable of degrading both soluble extracellular proteins and membrane proteins [48]. Although nano‐LYTACs lack inherent tissue specificity, which may result in reduced efficacy or off‐target effects, their LTR independence provides the potential to circumvent the “hook effect” [49].

Another limitation for PCSK9‐targeted LYTACs could be that PCSK9 is abundantly expressed, quickly cleared and reproduced, and rapidly regulated [50, 51]. In this case, frequent dosing of the LYTAC may be required, which could be prohibitive in real‐world applications. Nevertheless, LYTACs still hold the advantage of degrading extracellular, intracellular, and sporadically expressed proteins.

## 4 Experimental Section

### 4.1 Chemical Synthesis of Precursor Peptides

The LYTAC precursors were synthesized on a Symphony automatic synthesizer on Rink amide MBHA resin using Fmoc‐SPPS at a scale of 0.125 mmol. The coupling, Fmoc deprotection, and resin cleavage conditions are the same as reported in our previous work [17]. The azido‐alanine was incorporated into peptides using Fmoc‐β‐azido‐Ala‐OH as a building block. Crude peptides were purified by RP‐HPLC on a preparative C18 column (Phenomenex) with a 1%/min linear gradient from Solvent A (0.05% TFA, v/v) to 60% Solvent B (90% acetonitrile with 0.05% TFA, v/v in water) in Solvent A at a flow rate of 8 mL/min. The fractions containing the desired peptides, as determined by electrospray ionization mass spectrometry (ESI‐MS, Shimadzu), were pooled and lyophilized in a freeze‐drier.

### 4.2 Amine Acetylation of P9‐LYTAC3‐NGLH

The N‐terminal amine of P9‐LYTAC3‐NGLH was acetylated on resin. After the final coupling and removal of the Fmoc group, the resin was incubated with 6 mL of acetylation solution (DMF:DIPEA:acetic anhydride, 17:2:1, v/v/v) for 3 min, twice. After washing with DMF and DCM, the resin was dried in a desiccator. The peptide was then side‐chain deprotected and cleaved from the resin using the procedure described above.

### 4.3 Disulfide Bond Formation

The crude peptides were dissolved in 45% v/v acetonitrile in H_2_O at a concentration of 0.2 mg/mL, followed by the dropwise addition of 0.1 M I_2_ in acetonitrile until the solution turned a pale‐yellow color. The oxidation reaction was left in the dark at room temperature for 30 min. After quenching the reaction by the addition of ascorbic acid (until the solution turned back to transparent), the oxidized peptides were purified on a semiprep C18 column (Phenomenex) with a 0.5%/min linear gradient from 15% Solvent B to 45% Solvent B in Solvent A (v/v) at a flow rate of 3 mL/min.

### 4.4 Enzyme‐Assisted Azide Incorporation

The precursor peptide P9‐LYTAC3‐NGLH was dissolved in a ligation buffer consisting of 100 mM HEPES (4‐(2‐hydroxyethyl)piperazine‐1‐ethanesulfonic acid), pH 7.5. The ligation reaction consists of 100 µM of P9‐LYTAC3‐NGLH, 100 mM 3‐Azido‐1‐propanamine (1000 equiv.), 200 nM [C247A]OaAEP (0.005 equiv.), and ligation buffer at pH 7.5. The reaction was carried out with stirring at room temperature overnight. The ligation mixture was acidified by adding 5% TFA before purification via RP‐HPLC system (as mentioned above).

### 4.5 Copper‐Free Click Chemistry

The precursor peptides were dissolved in PBS buffer (137 mM NaCl, 2.7 mM KCl, 8.1 mM Na_2_HPO_4_, 1.5 mM KH_2_PO_4_, pH 7.4) at a concentration of 2 mg/mL, the same molar equivalent of DBCO–Tri‐GalNAc (Figure S3.1) was then added to the solution. The reaction mixture was incubated on a shaker (750 rpm) at 25°C for 2 h. Afterward, the mixture was acidified by adding TFA and loaded on a semiprep column and purified using a 0.4%/mL linear gradient from 25% to 40% Solvent B against Solvent A (v/v). The purity of ligated P9‐LYTACs was evaluated using a LC–MS system (Figure 4, Table S1).

### 4.6 NMR Spectroscopy

All NMR spectra were recorded at 298 K on a Bruker Avance 600 MHz spectrometer. P9‐LYTACs were dissolved in H_2_O:CD_3_CN (7:3. v/v) at 1 mg/mL concentration. The measured chemical shifts were referenced to sodium 3‐(trimethylsilyl)propane‐1‐sulfonate (DSS) at 0 ppm. TOCSY and NOESY were performed to calculate the secondary ^1^H chemical shift of the designed peptides compared to the random coil chemical shift values [37]. Spectra were analyzed using TopSpin and CCPNMR software packages [52].

### 4.7 SPR Binding Assays

SPR experiments were performed on a BIAcore T200 instrument on a SA chip (Cytiva) at 25°C. Biotinylated human PCSK9 (expressed and biotinylated by the Protein Expression Facility, The University of Queensland) was immobilized on a SA chip until a response of 1500 RU was achieved [17]. The peptides were dissolved in running buffer A (20 mM HEPES pH 7.4, 100 mM NaCl, 0.05% v/v Tween‐20) with a concentration of 324 nM and diluted threefold serially for four times. The peptide solutions were injected sequentially from the lowest concentration. The duration for each peptide injection on the channels was 120 s at a flow rate of 30 µL/min. A reference channel that contained streptavidin only was subtracted from the PCSK9‐immobilized channel. Data were analyzed with GE BIAevaluation software using a 1:1 binding model. The *K*_D_ values were the average ± standard deviation (SD) of triplicate.

Biotinylated human ASGPR (ACRO, GS1‐H82Q3‐25 μg) was immobilized on a SA chip until the response of 700 RU was achieved; the theoretical maximal response (*R*_max_) of P9‐LYTACs is 100, as calculated below. The peptides and Propylamine–Tri‐GalNAc were dissolved in running buffer B (20 mM HEPES pH 7.4, 100 mM NaCl, 5 mM CaCl_2_, 0.05% v/v Tween‐20) with a concentration of 1024 nM and diluted fourfold serially for four times. The following SPR method for measuring ASGPR binding affinity was the same as PCSK9.

$$R_{\max}=\frac{R_{\mathrm{ligand}}\times {\mathrm{Mr}}_{\text{analyte}}\times {\text{Valency}}_{\text{ligand}}}{Mr_{\text{ligand}}}\left(\mathrm{RU}\right)$$

Here, *R*_ligand_ is the response unit of the immobilized ASGPR, which is 700 RU. Mr_analyte_ is the molecular weight of P9‐LYTACs, approximately 4 kDa, Mr_ligand_ is the molecular weight of ASGPR, approximately 28 kDa. The valency of P9‐LYTACs binding to ASGPR is 1. Therefore, the theoretical *R*_max_ of P9‐LYTACs is 100 RU.

$$R_{\max}=\frac{700\left(\mathrm{RU}\right)\times 4\left(\mathrm{kDa}\right)\times 1}{28\left(\mathrm{kDa}\right)}=100\left(\mathrm{RU}\right)$$

### 4.8 Serum Stability Assay

Human serum (Sigma–Aldrich, H4522) was thawed and centrifuged (14,000 rpm at 4°C for 10 min) to remove lipids before incubating at 37°C for 15 min to activate the proteases. P9‐LYTACs were prepared as 300 µM stock solution in PBS and added to the activated serum in a ratio of 1:9 (v/v). The solution was incubated at 37°C for 48 h, and aliquots were removed after specific time points (0, 1, 2, 4, 8, 24, and 48 h). At each time point, 40 µL of the solution was removed from the incubator, mixed with 40 µL 6 M urea, and incubated for 10 min at 4°C. The mixture was mixed with 40 µL of 20% TCA and incubated for 10 min at 4°C. Finally, the quenched solutions were centrifuged at 14,000 rpm for 10 min at 4°C and 100 µL of the supernatant was loaded onto an analytical C18 column (Phenomenex) and analyzed by an HPLC system using a linear gradient from 25% to 55% v/v Solvent B in Solvent A. The percentage of the remaining peptide was quantified by measuring the peak area from the HPLC chromatograms at 214 nm at designated time points and normalized to the levels detected at 0 h. Peptides were tested in triplicate. The stability data of peptides were calculated with GraphPad Prism using nonlinear regression and a one‐phase decay model (single exponential).

### 4.9 Cell Culture and Treatment

Human hepatocellular carcinoma cell line HepG2 (RRID: CVCL_0027) was obtained from the Institute of Basic Medical Sciences, Chinese Academy of Medical Sciences (Beijing, China), and maintained in our laboratory. Cells were cultured in Dulbecco's Modified Eagle Medium (DMEM; Gibco, USA) supplemented with 10% fetal bovine serum (FBS; Gibco, USA) and 1% penicillin/streptomycin (Gibco, USA) at 37°C in a humidified atmosphere with 5% CO_2_.

### 4.10 Western Blotting Assay

Whole‐cell lysates were extracted from HepG2 cells with a Protein Extraction Reagent (Novagen) supplemented with a complete EDTA‐free protease inhibitor cocktail (Roche). Following separation on 10% SDS‐PAGE gels, proteins were transferred onto a PVDF membrane (Millipore, Bedford, MA, USA), which was then incubated with primary antibodies against PCSK9 (R&D Systems, Minneapolis, MN, USA), LDLR (Abcam, Cambridge, UK), or GAPDH (ZSJQ‐Bio, Beijing, China), followed by incubation with HRP‐conjugated secondary antibodies (ZSJQ‐Bio). Protein bands were visualized using an enhanced chemiluminescence detection system (Millipore) and quantified with ImageJ software, with GAPDH serving as the loading control for normalization.

### 4.11 Enzyme‐Linked Immunosorbent Assay

The concentration of sPCSK9 in the culture medium was measured using a commercially available ELISA kit (Solarbio) in accordance with the manufacturer's instructions. In brief, standards and samples were added to an antibody‐coated microplate specific for PCSK9. Following sequential incubation with a biotinylated detection antibody and streptavidin‐HRP, TMB substrate was added, and the absorbance was measured at 450 nm. The concentration of sPCSK9 was determined by reference to a standard curve.

### 4.12 Statistical Analysis

All experiments were repeated independently at least three times. All data are presented as the mean ± SD. Comparisons between two groups were analyzed using Student's *t*‐test. For comparisons among multiple groups, one‐way analysis of variance (ANOVA) with Bonferroni's post hoc test was applied. A two‐tailed *p* value < 0.05 was considered statistically significant. Sample sizes (*n*) and specific statistical details for each experiment are provided in the corresponding figure legends. Quantitative data from Western blot band intensity analysis were measured by densitometry using ImageJ software. Statistical analyses were performed using GraphPad Prism (version 9.0).

## 5 Supporting Information

Additional supporting information can be found online in the Supporting Information section. **Supporting** **Figure** **S1**: **Structure of** **Tri‐GalNAcs.** (A) DBCO–Tri‐GalNAc. (B) Propylamine–Tri‐GalNAc. **Supporting Figure S2: Comparison of the** **^1^** **H‐NMR spectra of P9‐** **LYTACs at 298 K**. In the amide region of P9‐LYTAC2, particularly the two tryptophan peaks around 10 ppm exhibited splitting, suggesting peptide isomerization and complicating the assignment of secondary chemical shifts. **Supporting Figure S3**: The affinity fitting curves of P9‐LYTACs binding to ASGPR. **Supporting Figure S4: Representative SPR** **sensorgrams for controls used in the ASGPR binding experiment**. (A) P9‐mut did not show any binding to ASGPR. (B) Propylamine–Tri‐GalNAc exhibited binding to ASGPR, with a binding peak observed between 760 and 900 s, corresponding to the injection at 1.024 µM. **Supporting Figure S5: Cellular PCSK9 degradation activity of P9‐** **LYTACs**. (A) ELISA analysis of the secreted PCSK9 levels in HepG2 cells treated with PBS, 20 or 200 nM P9‐LYTACs, and P9‐mut (data are shown as mean ± SD, n = 3). (B) Western blots of PCSK9, secreted PCSK9 (sPCSK9), and LDLR levels in HepG2 cells treated with 200 nM P9‐LYTACs or P9‐mut for 24 h. The data represent the mean ± SD of at least three independent experiments. *p < 0.05, **p < 0.01, ***p < 0.001 versus PBS control. **Supporting Figure S6: Western blots showing the levels of PCSK9 and LDLR in HepG2 cells treated with 10 µM C5 for 24 h.** Compared with PBS‐treated cells, cells treated with 10 µM C5 showed lower levels of secreted PCSK9 and higher levels of LDLR. The data represent the mean ± SD of at least three independent experiments. **p < 0.01, ***p < 0.001 vs. PBS control**.** **Supporting Table S1**: Peptides synthesized in this study.

## Funding

This work was supported by the National Health and Medical Research Council (GNT1107403 and GNT2009564) and the Australian Research Council (CE200100012).

## Conflicts of Interest

The authors declare no conflicts of interest.

## Supporting information

Supplementary Material

## Data Availability

The data that support the findings of this study are available in the supplementary material of this article.
