# Supplementary material for: Enzyme‐Assisted Synthesis and In Vitro Characterization of Bifunctional PCSK9 Inhibitors
Source: Chembiochem. 2026 Apr 8;27(7):e202500972. doi: 10.1002/cbic.202500972 (PMC13059054; doi:10.1002/cbic.202500972)
Supplement: Supplementary file 1 — Supplementary Material [file CBIC-27-e202500972-s001.pdf]

Enzyme-assisted synthesis and in vitro characterization of bifunctional PCSK9 inhibitors

Yuhui Zhang[†,1], Li Wang[†,2], Leo Corcilius[3], Amal K. Reji[1], Richard J. Payne[3], Bin Hong[2], Thomas Durek[1], Conan K. Wang[1], David J. Craik[1]

† These authors contributed equally

1 Institute for Molecular Bioscience, The University of Queensland, Brisbane, QLD 4072, Australia

2 Institute of Medicinal Biotechnology, Chinese Academy of Medical Science & Peking Union Medical College, Beijing 10050, China

3 School of Chemistry, The University of Sydney, Sydney, NSW 2006, Australia

Content

Figure S1. Structure of Tri-GalNAcs. 2

Figure S2. Comparison of the ^{1}H-NMR spectra of P9-LYTACs at 298 K. 2

Figure S3. The affinity fitting curves of P9-LYTACs binding to ASGPR. 3

Figure S4. Representative SPR sensorgrams for controls used in the ASGPR binding experiment 3

Table S1. Peptides synthesised in this study. 4

Figure S5. Cellular PCSK9 degradation activity of P9-LYTACs. 5

Figure S6. Western blots showing the levels of PCSK9 and LDLR in HepG2 cells treated with 10 µM C5 for 24 hours. 6

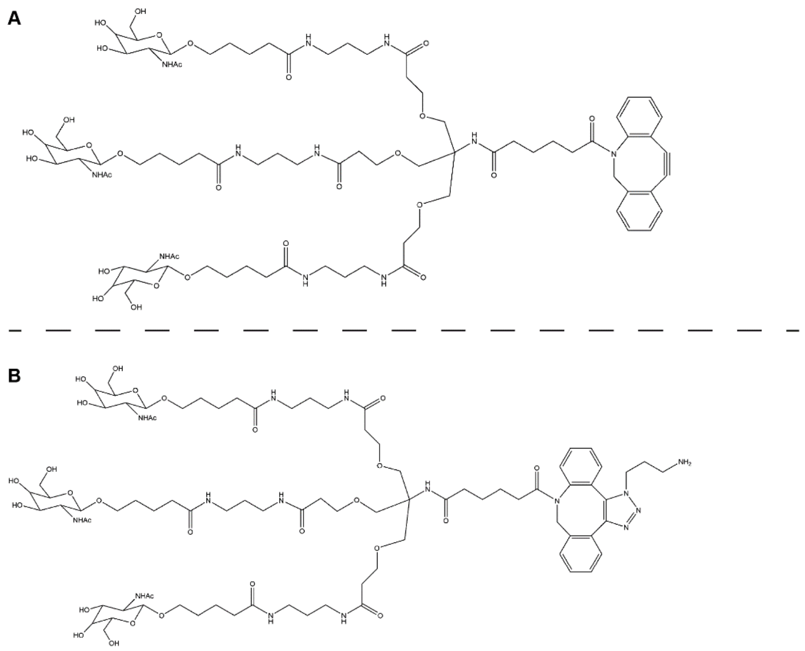

Figure S1. Structure of Tri-GalNAcs. (A) DBCO-Tri-GalNAc. (B) Propylamine-Tri-GalNAc.

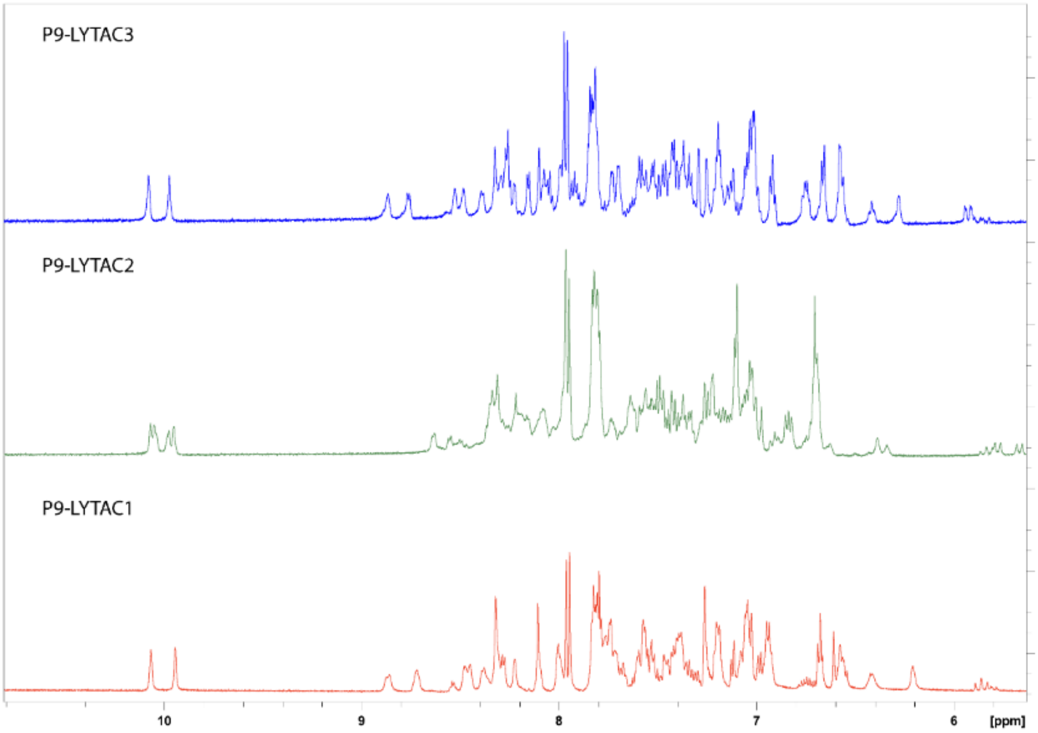

Figure S2. Comparison of the ^{1}H-NMR spectra of P9-LYTACs at 298 K. In the amide region of P9-LYTAC2, particularly the two tryptophan peaks around 10 ppm exhibited splitting, suggesting peptide isomerization and complicating the assignment of secondary chemical shifts.

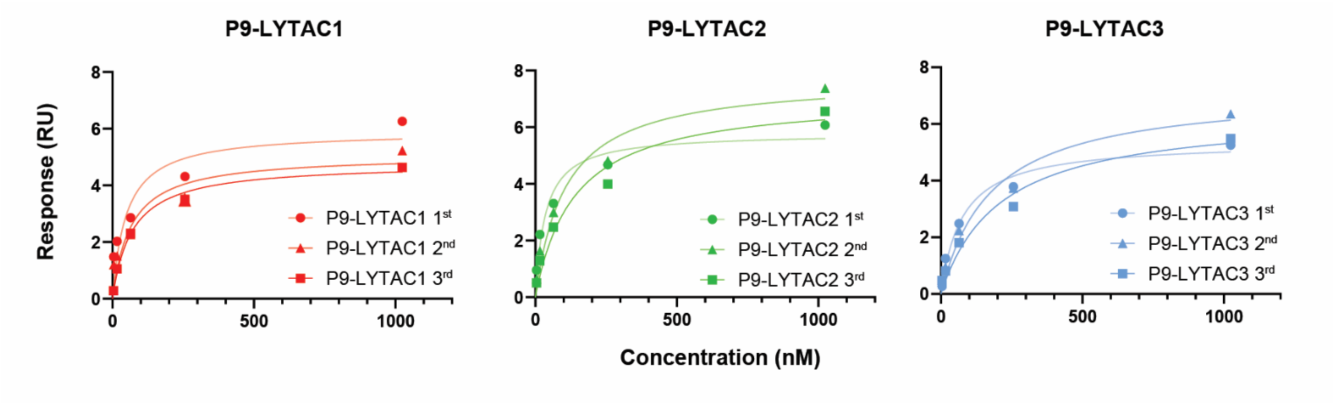

Figure S3. The affinity fitting curves of P9-LYTACs binding to ASGPR.

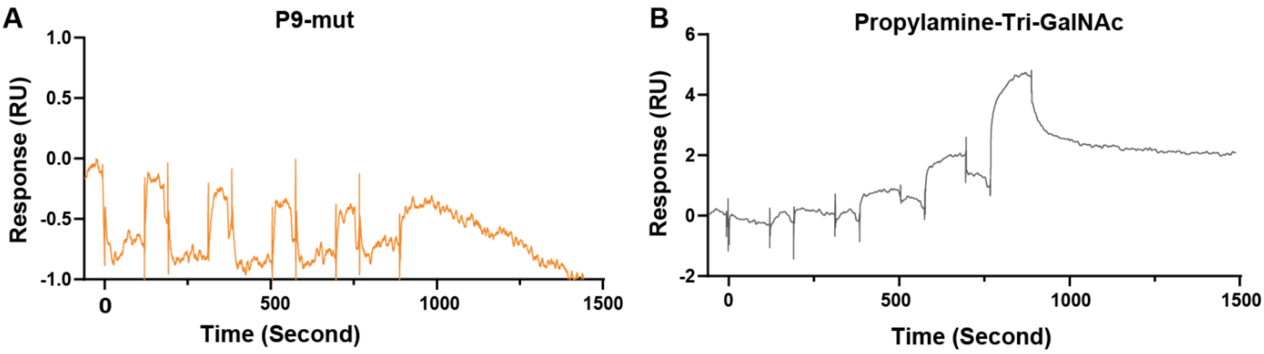

Figure S4. Representative SPR sensorgrams for controls used in the ASGPR binding experiment. (A) P9-mut did not show any binding to ASGPR. (B) Propylamine-Tri-GalNAc exhibited binding to ASGPR, with a binding peak observed between 760 and 900 seconds, corresponding to the injection at 1.024 µM.

Table S1. Peptides synthesised in this study.

Peptide	Sequence	Modification	Theoretical MW	Observed MW
**Pre-P9-LYTAC1**	122CTVFTSWEEYLDWNAKHPRNSC#	1=Ala(N_3)<br>2=PEG2<br>Cys cyclized	3086.4	3086.2 ± 0.2
**Pre-P9-LYTAC2**	CTVFTSWEEYLDWN1KHPRNSC#	1=Ala(N_3)<br>Cys cyclized	2725.0	2724.7 ± 0.3
**P9-LYTAC-NGL**	*CTVFTSWEEYLDWNAKHPRNSC22NGLH#	2=PEG2<br>Cys cyclized	3594.0	3593.7 ± 0.3
**Pre-P9-LYTAC3**	*CTVFTSWEEYLDWNAKHPRNSC22N-Azide	2=PEG2<br>Cys cyclized	3369.7	3369.4 ± 0.2
**P9-LYTAC1**	X22CTVFTSWEEYLDWNAKHPRNSC#	X=Ala(Tri-GalNAc)<br>2=PEG2<br>Cys cyclized	4817.3	4816.9 ± 0.1
**P9-LYTAC2**	CTVFTSWEEYLDWNXKHPRNSC#	X=Ala(Tri-GalNAc)<br>Cys cyclized	4455.9	4455.5 ± 0.2
**P9-LYTAC3**	*CTVFTSWEEYLDWNAKHPRNSC22N-TriGalNAc	2=PEG2<br>Cys cyclized	5100.6	5100.2 ± 0.3

C-terminal amidation, *N-terminal acetylation. The theoretical molecular weight (MW) is calculated by ChemDraw, and the calculated MW is based on the peptide’s m/z values of ions.

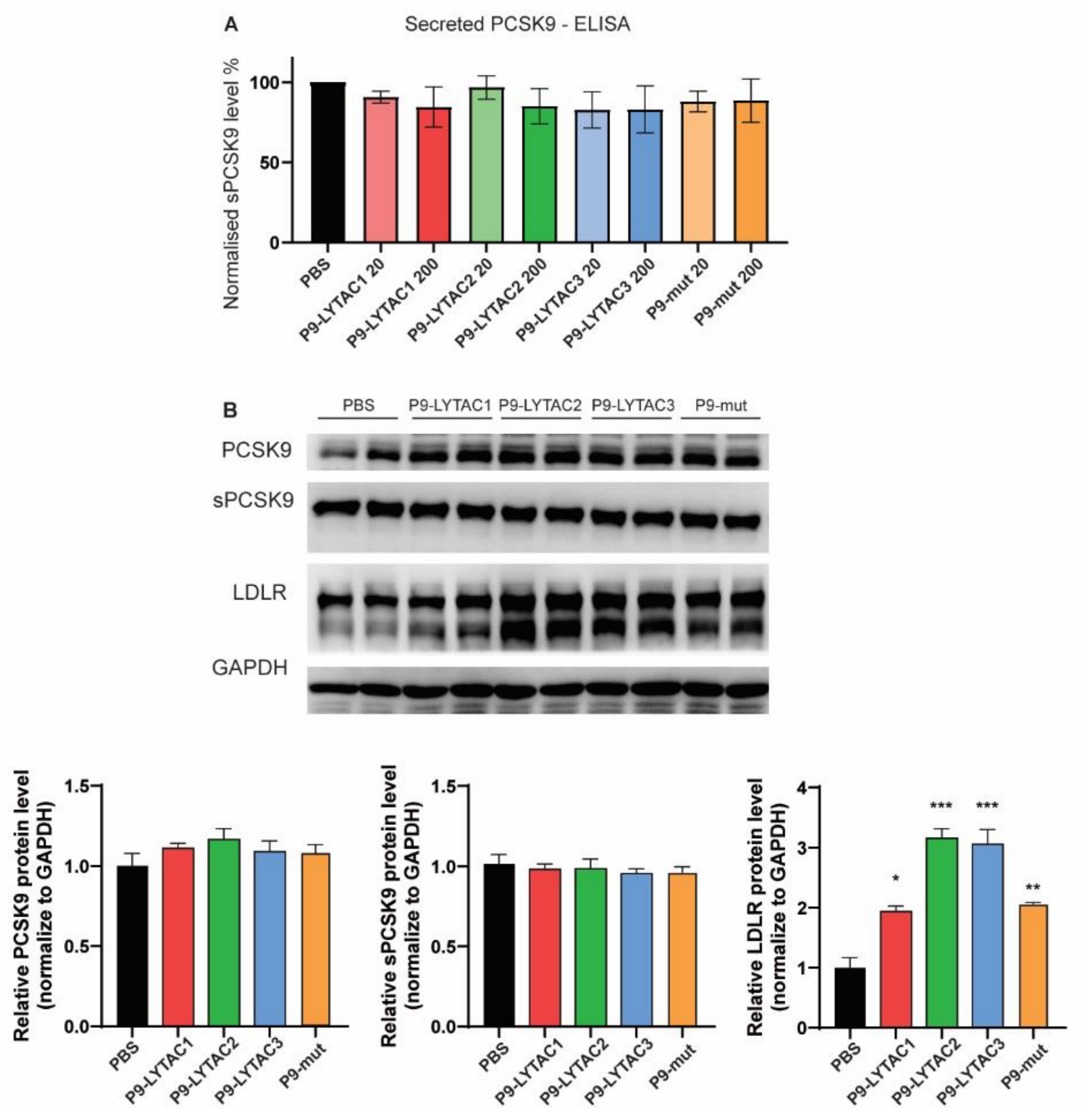

Figure S5. Cellular PCSK9 degradation activity of P9-LYTACs. (A) ELISA analysis of the secreted PCSK9 levels in HepG2 cells treated with PBS, 20 or 200 nM P9-LYTACs, and P9-mut (data are shown as mean ± SD, n = 3). (B) Western blots of PCSK9, secreted PCSK9 (sPCSK9), and LDLR levels in HepG2 cells treated with 200 nM P9-LYTACs or P9-mut for 24 h. The data represent the mean ± SD of at least three independent experiments. *$p < 0.05$, **$p < 0.01$, ***$p < 0.001$ vs. PBS control.

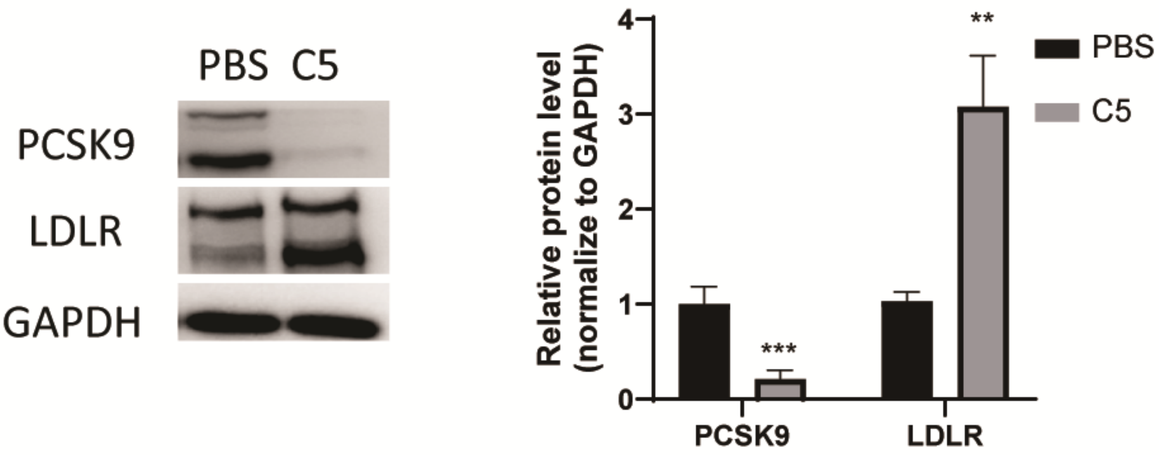

Figure S6. Western blots showing the levels of PCSK9 and LDLR in HepG2 cells treated with 10 µM C5 for 24 hours. Compared with PBS-treated cells, cells treated with 10 µM C5 showed lower levels of secreted PCSK9 and higher levels of LDLR. The data represent the mean ± SD of at least three independent experiments. **$p < 0.01$, ***$p < 0.001$ vs. PBS control.
